# Supplementary material for: COVCOG 2: Cognitive and Memory Deficits in Long COVID: A Second Publication From the COVID and Cognition Study
Source: Front Aging Neurosci. 2022 Mar 17;14:804937. doi: 10.3389/fnagi.2022.804937 (PMC8967943; doi:10.3389/fnagi.2022.804937)
Supplement: Supplementary file 1 [file Data_Sheet_1.docx]

COVCOG 2: Cognitive and Memory Deficits in Long COVID:
A Second Publication from the COVID and Cognition Study.

Supplementary Material

**Supplementary Table 1.** Rotated component matrix for cognitive task variables.

| Rotated component matrix^a^ | Component | |
| --- | --- | --- |
|  | **EF** | **RT** |
| WCST_Correct | 0.941 | -0.103 |
| WCST_Persev_error | -0.888 | 0.162 |
| WCST_Nonpersev_error | -0.627 | -0.131 |
| WCST_RT_correct | -0.278 | 0.807 |
| WCST_RT_Nonpersev | 0.032 | 0.563 |
| @2D_CorrectPercentage | 0.585 | 0.111 |
| @2D_RT_overall | 0.252 | 0.734 |
|  | **Memory** | **Category Fluency** |
| WL_CorrectPercentage | 0.84 | 0.116 |
| WL_RT_overall | -0.501 | 0.194 |
| Category_correct | 0.484 | 0.446 |
| Cetagory_related | 0.172 | -0.722 |
| Category_incorrect | -0.025 | -0.661 |
| Category_CorrectPercentage | 0.114 | 0.885 |
| AM_correctPercentage | 0.475 | 0.216 |
| AM_RT_overall | -0.445 | 0.192 |
| WL_d | 0.816 | 0.069 |
| Extraction Method: Principal Component Analysis. | | |
| Rotation Method: Varimax with Kaiser Normalization. | | |
| ^a^ Rotation converged in 3 iterations. | |  |

**Supplementary Table 2.** Group and pairwise comparisons including both No COVID and severity levels for cognitive factors.

| **Comparing No COVID, Recovered, Ongoing (Mild/Moderate) and Ongoing (Severe) groups** | | | | |
| --- | --- | --- | --- | --- |
|  | **Primary (uncontrolled) comparison** | | **Comparison controlling for age, sex, country and education level (GLM)** | |
|  | ***T*/*F* (df)** | ***p*** | ***F* (df)** | ***p*** |
| **Factor 1:  EF performance** | 1.318 (3,317) | .268 | 0.524 (3,303) | .666 |
| **Factor 2:  EF Reaction Time** | 3.719 (3,307) | .012* | 1.422 (3,293) | .236 |
| **Factor 3: Memory** | 6.688 (3,304) | <.001*** | 8.453 (3,290) | <.001*** |
| **Factor 4:  Category Fluency** | 2.707 (3,313) | .045* | 0.982 (3,288) | .402 |
| **Pairwise Comparisons** | | | | |
| **Factor 3: Memory** | **No COVID** | **Recovered** | **Mild/Moderate** | **Severe** |
| **No COVID** | - | ns | *t*(87.6) = 2.4, *p* = .018 | *t*(99.8) = 3.9, *p* < .001 |
| **Recovered** | - | - | ns | *t*(99) = 2.885, *p* = .005 |
| **Mild/Moderate** | - | - | - | ns |
| **Severe** | - | - | - | - |
| **Factor 4:  Category Fluency** | **No COVID** | **Recovered** | **Mild/Moderate** | **Severe** |
| **No COVID** | - | ns | ns | *F*(1,152) = 3.051, *p* = .003 |
| **Recovered** | - | - | ns | ns |
| **Mild/Moderate** | - | - | ns | ns |
| **Severe** | - | - | - | - |
| **Pairwise Comparisons Controlling for Age, Sex, Country and Education** | | | | |
| **Factor 3: Memory** | **No COVID** | **Recovered** | **Mild/Moderate** | **Severe** |
| **No COVID** | - | ns | *F*(1,191) = 5.515, *p* = .02 | *F*(1,205) = 20.38, *p* < .001 |
| **Recovered** | - | - | ns | *F*(1,85) = 6.65, *p* = .012 |
| **Mild/Moderate** | - | - | - | ns |
| **Severe** | - | - | - | - |

**Supplementary Table 3.** Initial phase neurological symptoms and subsequent cognitive performance.

| **Symptom Factor  (Predictor)** | **Cognitive Outcome (dependent variable)** | ***F*** | ***p*** | ***β*** | **Adjusted *R^2^*** |
| --- | --- | --- | --- | --- | --- |
| **Headache** | Word List (RT) | (1,158) = 7.92 | .005 | -.219 | .042 |
|  | Associative Memory  (% Correct) | (1,159) = 4.48 | .036 | .165 | .021 |
|  | Category Fluency (Correct) | (1,156) = 8.87 | .003 | .0232 | .048 |
| **Confusion** | Category Fluency  (% Correct) | (1,155) = 4.00 | .047 | .159 | .019 |
|  | Word List (% Correct) | (1,158) = 7.83 | .006 | .217 | .047 |
| **Altered Consciousness** | Word List (d’) | (1,158) = 9.14 | .003 | .234 | .049 |
| **Dizziness** | WCST (persev. errors) | (1,156) = 4.54 | .035 | -.168 | .028 |
| **Disorientation** | WCST (Correct) | (1,156) = 5.57 | .019 | .186 | .034 |
| **Numbness** | WCST (RT Correct) | (1,156) = 5.57 | .003 | -.234 | .055 |

* denotes *p* values below Sidak-correct alpha at .0028
